# “My Favourite Day Is Sunday”: Community Perceptions of (Drug-Resistant) Tuberculosis and Ambulatory Tuberculosis Care in Kara Suu District, Osh Province, Kyrgyzstan

**DOI:** 10.1371/journal.pone.0152283

**Published:** 2016-03-28

**Authors:** Doris Burtscher, Rafael Van den Bergh, Ulan Toktosunov, Nilza Angmo, Nazgul Samieva, Eva P. Rocillo Arechaga

**Affiliations:** 1 Médecins Sans Frontières, Vienna Evaluation Unit, Vienna, Austria; 2 Médecins Sans Frontières, Brussels, Belgium; 3 Ministry of Health, Bishkek, Kyrgyzstan; 4 Médecins Sans Frontières, Bishkek, Kyrgyzstan; 5 Médecins Sans Frontières, Osh, Kyrgyzstan; St. Petersburg Pasteur Institute, RUSSIAN FEDERATION

## Abstract

**Objectives:**

Kyrgyzstan is one of the 27 high multidrug-resistant tuberculosis (MDR-TB) burden countries listed by the WHO. In 2012, Médecins Sans Frontières (MSF) started a drug-resistant tuberculosis (DR-TB) project in Kara Suu District. A qualitative study was undertaken to understand the perception of TB and DR-TB in order to improve the effectiveness and acceptance of the MSF intervention and to support advocacy strategies for an ambulatory model of care.

**Methods:**

This paper reports findings from 63 interviews with patients, caregivers, health care providers and members of communities. Data was analysed using a qualitative content analysis. Validation was ensured by triangulation and a ‘thick’ description of the research context, and by presenting deviant cases.

**Results:**

Findings show that the general population interprets TB as the ‘lungs having a cold’ or as a ‘family disease’ rather than as an infectious illness. From their perspective, individuals facing poor living conditions are more likely to get TB than wealthier people. Vulnerable groups such as drug and alcohol users, homeless persons, ethnic minorities and young women face barriers in accessing health care. As also reported in other publications, TB is highly stigmatised and possible side effects of the long treatment course are seen as unbearable; therefore, people only turn to public health care quite late. Most patients prefer ambulatory treatment because of the much needed emotional support from their social environment, which positively impacts treatment concordance. Health care providers favour inpatient treatment only for a better monitoring of side effects. Health staff increasingly acknowledges the central role they play in supporting DR-TB patients, and the importance of assuming a more empathic attitude.

**Conclusions:**

Health promotion activities should aim at improving knowledge on TB and DR-TB, reducing stigma, and fostering the inclusion of vulnerable populations. Health seeking delays and adherence problems will be countered by further implementation of shortened treatment regimens. An ambulatory model of care is proposed when convenient for the patient; hospitalisation is favoured only when seen as more appropriate for the respective individual.

## Introduction

Each year, more than nine million people develop active TB, and nearly two million TB patients die. Multidrug-resistant TB (MDR-TB) and extensively drug-resistant TB (XDR-TB) are major threats to TB control, and all countries can be considered at risk of developing resistant TB. The standard six-month treatment with first-line anti-TB drugs is not effective for people with MDR-TB and XDR-TB. Instead, they must be treated with drugs that are less efficacious, more toxic and much more costly (typically, US$ 2000–5000 per patient). The duration of treatment is up to two years [1].

TB programmes are hampered considerably by the stigma surrounding TB, which has been shown to introduce delays to diagnosis and to reduce treatment adherence [2, 3], further enhancing the development and spread of resistant strains of TB. The countries of the former Soviet Union may be particularly vulnerable in this regard: they bear a high burden of TB (most are among the 27 high-burden countries for TB) [4], stigmatisation of TB has been common in the region [5, 6], and additionally, they have seen a sharp increase in the use of complementary and alternative medicine, which may complicate the uptake of new models of care for TB control [7].

Kyrgyzstan is one of these vulnerable countries, facing the multi-pronged challenge of a high TB burden, stigma, prevalent drug and alcohol abuse and addiction, presence of neglected groups as well as migrants and homeless people facing barriers in accessing health care facilities, and a relatively widespread use of either hospitalised models of care or alternative medicine, leaving little space for new models of care.

In this setting, the non-governmental organisation Médecins Sans Frontières (MSF), in collaboration with the Ministry of Health (MoH), started a DR-TB programme in Kara Suu District, relying on a patient-centred approach focusing on ambulatory TB treatment, intensive case finding and comprehensive DR-TB management. During the preparation and initial phases of this programme, several difficulties were encountered. In addition to technical challenges to the provision of care, extensively described in Central Asia in the scientific literature [8–15], difficulties related to TB and DR-TB perception were also found, such as limited new cases from high risk groups, high treatment refusal rates, strong resistance of community members to ambulatory services, and a perception of mismanagement by health care providers.

A number of studies have provided insight into the region’s core traditional belief systems [9] and the use of complementary and alternative medicine [7]. The TB health seeking behaviour in several Asian contexts and elsewhere has also been examined previously [8, 10–14]. However, considerable regional variation may exist in beliefs and practices around TB, which remain largely unmapped. Additionally, the extent to which populations’ perceptions of the disease and provision of treatment influences the uptake of novel models of care, in particular in regions with a considerable presence of alternative health care providers, has remained unexplored. We therefore conducted a qualitative study on the perceptions of TB among patients, caretakers, health care providers, and community members in Kyrgyzstan. The specific objectives were to assess the perceptions and acceptability of an ambulatory model of care for DR-TB, as recommended by the WHO [15, 16].

## Methods

### Study setting

This qualitative study took place in the Kara Suu District of Osh Province in the south of Kyrgyzstan. Osh Province, with just over a million inhabitants, contributed up to 25% of the TB cases detected in the country in 2013 (1470 out of a total of 6082 TB cases). The estimated TB incidence in Kyrgyzstan in 2013 was 141/100,000 persons, prevalence was 190/100,000 and mortality 11/100,000. Alarmingly high levels of MDR-TB were reported by the WHO among new cases (26%) and TB re-treatment cases (55%) [4]. HIV prevalence in adults (15–49 years) is 0.7% and IDU prevalence is 14.6% according to the Global Fund report [17].

TB services in Kara Suu District are provided mainly through a TB hospital with inpatient care. Since 2012, the MoH and MSF have provided ambulatory care through 108 peripheral facilities including three TB cabinets with TB doctors and nurses, 27 Family Group Practitioner Centres (FGP) with general practitioners, and 78 feldsher points with a nurse or a feldsher (a position between doctor and nurse). Until the end of 2014, a total of 300 patients were enrolled in treatment (67 in 2012, 131 in 2013 and 102 in 2014).

### Study methodology

A qualitative research design was employed, using an exploratory approach to understand the knowledge and perceptions of TB and DR-TB from the perspective of the general population, patients and care providers (doctors and nurses) in Kara Suu District [18]. A flexible participatory technique was applied, i.e. the researcher gathered data using non-participant observation, field notes as well as in-depth interviews guided by topic-led questions [19]. The participatory approach included working directly with the affected people as they are considered knowledgeable specialists of their own experiences regarding TB and DR-TB; they have first-hand knowledge and therefore participated in the study [20]. The questions were based on themes relevant to the research question and literature search (literature review in S1 File). Following standard qualitative interview procedures, the order of questions was driven by the nature of each participant’s answers, i.e. both the wording of questions and the order they were asked during interviews were likely to be modified. With specific vulnerable individuals, focus group discussions were conducted. A triangulation of findings, using multiple methods or perspectives for data collection, was undertaken to enhance the interpretation of data [21]. A methodological triangulation was applied: different methods were used throughout the study process, including tools (document reviews and literature search prior to the fieldwork; in-depth individual interviews, one group discussion, several focus group discussions, non-participant observations); time (e.g. during/after treatment intake); space (at home, in the clinic or in the hospital); and study subjects (patients, caretakers/health care providers) [22, 23].

### Study population

Six main groups of individuals were included in the study (please refer to Table 1):

1. General population and neglected groups in the area of intervention
2. Patients under treatment or having completed TB and DR-TB treatment
3. Patients who stopped or refused TB and DR-TB treatment
4. Health care providers dealing with TB and DR-TB patients and other general health care providers
5. Family members and caregivers of TB and DR-TB patients
6. Other alternative health care providers (healers, priests, etc.) who were visited by patients

**Table 1:** Interview characteristics, Kara Suu District and Osh City, Kyrgyzstan, January-February 2014.

Interview type	Total interviews = 63; n (%)	
Individual interview	58	(92)
Focus Group Discussion	4	(6)
Group Interview	1	(2)
**Interviewee profile**		
Patients	18	(28)
Caregivers and family members	8	(13)
Neglected groups (not MSF and not TB patients)	7	(12)
Health care providers (16 MoH/2 MSF)	18	(28)
MSF staff	7	(11)
General population	5	(8)

The study engaged in purposive sampling. Consenting informants were selected based on the criteria that they were infected with DR-TB and received treatment (by MSF and the MoH). Patients who were not able to start treatment were also interviewed. Additionally, family members and caretakers as well as health care personnel (both MoH and MSF) and the general population of the area were part of the study. Members of the general population were identified with the help of a social worker or village head. Additionally, study participants were selected based on age, gender, religion and ethnicity; patients were also selected according to the criteria of their in/outpatient status, treatment uptake, and treatment outcome; in order to ensure representation of perspectives from a range of groups in a diverse population. Individuals unable to provide consent, individuals under the age of 12, and individuals deemed too sick to give an interview were excluded.

All study participants were interviewed about their knowledge and perception of TB and DR-TB and the stigma surrounding TB. Additionally, health care providers were asked about their experience and perception of working in a health care structure dealing with TB patients, from their own point of view and as viewed by the general population and their families. Patients were also asked about the health care provided to them, their treatment experience, treatment support and perception of their social environment, health seeking behaviour, barriers to health care, perception of the health structures in general, and perception of the MSF services.

### Data collection

For data collection related to the knowledge and perception of TB and DR-TB, in-depth individual interviews were conducted. This qualitative method provides an emic perspective of people who are suffering from TB or DR-TB, of families who care for a TB patient and of the population in general, which is also affected by TB. In addition to these target groups, in-depth interviews with health care providers were carried out. The researcher followed a topic guide of open-ended questions. These were structured to build trust and rapport, encourage openness and honesty of respondents, with more emotive questions brought up later in the interview. The topic guide was kept flexible to prevent the conversation from becoming too ‘vertical’. The researcher followed up on the answers and information the interviewees provided.

Data collection took place from January to February 2014. Fifty-eight in-depth individual interviews were conducted, four FGD and one group interview using a flexible topic guide allowing open-ended questions. Interviews were conducted in English and orally translated either into Kyrgyz, Uzbek, Russian or Luli language. The majority of interviews were recorded (48) or notes were taken (15) and then transcribed into English. Data were collected until no additional insights related to the research questions were gained (thematic saturation) [22, 24].

### Data analysis

Data were constantly reviewed and emerging themes related to the original research question as well as new areas were taken into consideration for further interviews. Coding was done manually. Data transcripts were analysed in an inductive and deductive way, using a qualitative content analysis [25]. The content was analysed in two ways: descriptively by describing data without reading anything into it and interpretatively by focusing on what might be meant by the responses [26]. All transcripts were screened for relevant information, and then organised, coded, categorised and interpreted. Codes were also developed based on anthropological theory known prior to the research. Anthropological theoretical concepts that occurred as a code were treatment-taking behaviour, adherence/concordance, illness perception, side effects, perception of recovery, health seeking behaviour and moralisation of TB, etc. [27]. Validation of data was ensured by triangulation and a ‘thick’ description of the research context, and by presenting deviant cases [28].

### Ethics

Ethical approval was granted by the Committee of Bioethics at the MoH in Bishkek, Kyrgyzstan, and by the Ethical Review Board of MSF. Written informed consent was obtained from all participants; in one case, the consent was given orally, and this was recorded. Efforts were made to avoid that their participation in this research would identify the interviewees as TB cases and thus increase their potential burden. All interviewed patients, caregivers and patients’ relatives were called and asked beforehand if they agreed to an interview and where and when they preferred to be interviewed. Inpatients were interviewed in Joosh TB hospital; outpatients were interviewed either in their homes or at one of the clinics were they received ambulatory treatment. Interviews were conducted in a confidential environment. At the request of some patients, an unmarked car (without MSF identification) was used when visiting them at home.

## Results

### Knowledge and perception of TB and DR-TB

The level of knowledge, understanding and awareness varied considerably between the different groups. Both for health care workers and in the general population, poor knowledge and awareness were noted in individuals who had never been in contact with anyone suffering from TB, while individuals who had been exposed to TB or DR-TB had a more concrete understanding of the disease, its symptoms, transmission pathways, preventive measures and treatment.

#### TB as a ‘dangerous’ disease causing ‘nervousness’

TB was perceived as a dangerous disease that people were afraid of. In some cases, doctors and nurses did not inform the patients about their correct diagnosis of DS-TB or DR-TB as it would stress them, and this would not be appropriate in the mentality of the Kyrgyz people.

“According to the mentality it is not so good to tell the people that they have TB, I am a Muslim, it is not polite, the person can be stressed and distressed, I don’t want to put people in this condition.” (female feldsher, 48 years)

Many patients had difficulties to accept that they were suffering from TB because of the stigma, but also because they did not understand why the disease had happened to them. Respondents repeatedly referred to feelings of tension and ‘nervousness’ when they talked about TB.

“Usually when the patients get diagnosed, they immediately get stressed; just when they hear that they have TB, they get nervousness. But after they come to the hospital they see other patients, they get used to that and that stress disappears little by little.” (female nurse, 41 years)

The perception of TB as a dangerous disease and the fact that doctors and nurses earn more when working in TB facilities fosters the notion of TB being a threatening condition, which adds to the stigmatisation of TB patients.

#### ‘The lungs catch a cold’

TB was self-diagnosed as a flu or (chronic) bronchitis, and was attributed to the concept of ‘cold disease’. It was seen as a disease of the lungs, ‘sticking’ inside the lungs, the lungs having holes or the ‘lungs catch[ing] a cold’. A man diagnosed with TB was convinced that he had a ‘cold of the lungs’ and not TB.

“We never had a history of lung diseases in our ancestors.

Yes, I and my wife, we do not have parents and grandparents who had lung diseases. Our children and children of our relatives are clean. It was just with me, but it was because I was caught in the heavy rain and wind in Jailoo [Jailoo is a summer pasture, an ancient tradition of nomadic pastoralism in Kyrgyzstan], that is why my lungs got sick by catching cold.” (male household member, 50 years)

Doctors did not talk openly to patients about TB, but spoke about an ‘inflammation of the lungs’.

“Usually our doctors also do not want to tell ‘you have TB’, they try to say it softly and say that the patient has ‘lungs inflammation’, and many patients are assured that they have inflammation of the lungs.

Why do you think they do not say it openly?

Sometimes doctors do this so that the patient will not stop the treatment. Our mentality is that if doctors prescribe something then the patient will take all the prescriptions. If the doctor will present the disease as an ‘inflammation of lungs’, and often the patients will not even suspect they have TB. I knew one woman who had TB but always she was telling that her lungs catch a cold.” (female social worker, 40 years)

#### Tuberculosis as a ‘chronic disease’

The widespread belief that TB is a chronic disease was linked to the assumption that TB is incurable. Many patients with DR-TB had a long anamnesis, dating back many years. They recounted being treated every year, and for many of these individuals it became a normal procedure and part of their life to treat ‘chronic TB’.

“Before going to the Osh hospital I had annual treatment every winter and every summer. I was working in the field again. I knew nothing about the disease, but I believed to the doctors, they know more than I do. I accepted that I am sick.

You said that you have been taking treatment every winter, was it TB treatment?

Yes.

How long during winter?

3 to 4 months (patient’s wife).

With the MoH [ministry of health] I was spending 1 or 1.5 months. The longest one was in Kara Suu, which was 3.5 months.

Was it always for TB?

Yes, always for TB.” (male patient, 58 years)

### Understanding of transmission

There was a notion that TB resides *in* the family and *in* the genes, and that it transmits from generation to generation. This was particularly the case when several family members suffered from the disease.

“It [TB] is transmitted from genes, blood, from generation to generation, if there was a person in the family who had been sick with TB, his/her children will be TB too without an exception.” (male household member, 50 years)

Another widespread belief was that TB is transmitted in crowded or unclean places, and the misconception that one can get contaminated by using the same dishes or clothes, by shaking hands with an infected person, or by kissing, was still prevalent. Additionally, some patients mentioned that TB is transmitted through sexual intercourse and/or through drug use.

Some health care providers, patients and their caregivers knew that TB can be transmitted by sitting close to an infected person, by staying in the same room as a TB case, or by coughing, sneezing and spitting. Well-informed patients knew that the infection comes from another sick person, but many still mentioned that they thought it was in their family and that they have the disease in the blood or inside the body.

“I think I was not infected (from another person); this disease was created in my body because I was suffering a lot, we had bad life conditions and usually I was hungry, I suffered a lot and I had a lot of problems. It is a self-born disease from inside my body. I could not use the drugs, I was in depression and was very nervous and I was vomiting and I think because I was nervous. I had a lot of worries in my life, it was a hard hit on my health that I got sick.” (female DR-TB patient, 41 years)

Interviews with patients showed that ‘blaming’ another person for the infection was not considered appropriate.

### Health seeking behaviour

The pathway of an individual to treatment usually starts with a ‘diagnosis’ inside the family, taken in charge by the eldest male in the family. Subsequently, the condition of the sick person needs to be defined and the causes reflected upon in order to decide how to respond, followed by the decision on where to go to get treatment. In most cases, a sick person was first treated at home, as most people think they suffer from the flu or a cold. Only when people did not recover did they go to a polyclinic, TB cabinet, feldsher point or to a hospital to see a doctor. In some occasions, they went to see a traditional healer, sometimes even with an already confirmed TB diagnosis.

Alternative ways of treatment were limited to a number of traditional health care providers, the *Moldo* and the *Tabib*. *Moldo* (the one who prays) is a religious man or woman who prays over the patient. Many participants specified that they do not go to see the *Moldo* for TB, but findings suggest that they play an important peripheral role in the psychological wellbeing of TB patients, and are responsible for a delay in seeking timely medical advice. The *Tabib* (Arabic for doctor) uses medication together with traditional and Islamic remedies. Respondents reported that some *Tabibs* are retired ‘doctors’ or feldsher.

### Treatment

#### Doctor/nurse-patient relationship

Since DR-TB requires long treatment, patients and health care providers engage in a longer-term relationship with the patient. They see the patient almost every day during a period of months in case of inpatients, and up to two years for outpatients. MoH health staff emphasised that they realised how important their role is, and the positive attitude of health staff was confirmed by patient reports.

“I like the attitude of nurses and doctors. That is all.

Which attitude do they show?

While you are suffering with the treatment they are following with polite words nice attitude. I am really grateful for their attention because even relatives cannot support like this.” (male inpatient, 45 years)

Numerous respondents expressed that they liked the MoH and MSF staff, including social workers, because “[…] they treat me very nice” (male inpatient, 61 years). Social workers from the patient support, education and counselling (PSEC) team play a central role in the support of in- and outpatients, as they can be called at any time for psychosocial support and provide encouragement to continue the treatment.

“For what reasons do they call for example?

They can tell about the side effects, if they have financial problems, if they are tired of the treatment saying ‘I can’t do it’. Sometimes we have long conversations, we deliver education sessions or when they lack coping skills like ‘I can’t do it. I understand that I need, but I can’t’.” (female social worker, 28 years old)

#### Inpatient versus outpatient treatment

The preference of inpatient versus outpatient care depended on the role of the person within the family (age, gender, hierarchy), the ethnical background, and the distance to the home village (associated with costs).

#### Inpatient preference

was mentioned by patients who were alone and had nobody at home to care for them, as inpatient care would bring companionship. Additionally, inpatient care represented safety (proximity to health staff) as well as a means to avoid daily transport–in particular in the context of DR-TB treatment, which due to the severe side-effects necessitates rest after drug intake.

#### Outpatient preference

was mentioned by most other participants. Young males preferred ambulatory care as they could see their friends, be with their families, and have more diversity in their lives. Family members expressed a preference for ambulatory treatment as they could provide better emotional support this way. Patients frequently mentioned that encouraging words from their families helped most, along with financial support when necessary. Patients experiencing stigma (HIV, drug/alcohol users, ex-prisoners) also preferred ambulatory care; similarly, some patients wished to avoid hospitalisation in the same facility as ex-prisoners, drug/alcohol users, HIV-positive patients and the homeless.

Health staff opinions were also divided: for patients with family support and patients experiencing stigma, ambulatory care was considered best, while inpatient care was preferred for patients suffering from strong side effects, in order to be able to monitor and deal with the side effects and motivate the patient to continue the treatment.

#### Factors influencing treatment concordance

Four major aspects influencing treatment behaviour were identified:

- structural factors (often constraining the agency of the patient and negatively influencing the patients’ ability to act),
- social context (emotional and financial support by families, friends and neighbours), including poverty and gender discrimination (young women and *kelin* [daughter in law])
- health service factors (trust in the health structure, staff’s attitude)
- personal factors (knowledge about the disease, side effects and length; pregnancy; family)

All medical staff, MoH and MSF, emphasised that treatment is easier to tolerate for young people in terms of side effects, but that they are less likely to complete the treatment. In contrast, older people usually have no problem with stigma, but suffer from more severe side effects, which negatively impact their health and lead to treatment interruption/cessation.

### Stigma and access barriers

Nearly all TB patients spoke of experiencing stigma or discrimination in one way or another. Neighbours and relatives stopped visiting, they did not get a job or felt that people ‘avoided’ them. Some patients also experienced stigma from health staff, and described this as even more disappointing, as they expected health staff to have a professional understanding of TB. Young women particularly suffered from stigma: in Uzbek and Kyrgyz tradition, the selection of the daughter-in-law is made according to her family background, good health and strong physical condition. When she suffers from ill health, she loses her ‘significance’ and is considered inappropriate for the role of a daughter-in-law. Families can try to separate the couple when they know that the daughter-in-law has TB, even against the will of the husband.

“If the young bride got sick, husband’s family come to us with question and ask whether they need to get divorced, if it is very dangerous, etc. We had many of this kind of situations. There were many cases where the couple was reunited, but there were also many couples that got separated too.” (female nurse MoH, 60 years)

As a consequence of the fear of being rejected by the family-in-law or of being separated from the husband/never finding a husband, numerous young women diagnosed with TB do not start treatment. Other vulnerable groups in terms of stigma included the homeless, drug/alcohol users, migrants returning from Russia (who cannot access health care in Russia), and people from the Luli community (due to general poor living conditions and poverty).

## Discussion

Our study represented one of the first analyses of the perceptions and opinions on tuberculosis and TB therapy among people affected by TB in Kyrgyzstan: although extensive research has addressed clinical and medical aspects of TB and DR-TB in Central Asia [29–36], the social aspects and perception of TB and DR-TB in the region and especially in Kyrgyzstan remain unexplored in the scientific literature. Additionally, this study represents one of the first assessments of patient perception/preference of ambulatory versus inpatient care. Perceptions of TB and DR-TB were complex and led to various consequences, such as delays in diagnosis and treatment initiation.

The novel finding of TB being interpreted as a ‘cold disease’ has a major impact on how people deal with the illness. People manage first signs and symptoms in ways that disguise TB and delay effective treatment. As doctors do not always talk openly to patients about TB infection, the notion of TB as a ‘cold disease’ may have been communicated by health personnel and later adopted as a concept by patients and the general population. At the same time, this concept helps address the fear of stigma–as suffering from a ‘cold’ or ‘inflammation of the lungs’ is not as stigmatising as having TB. Similarly, patients accept the idea that TB is inherited and a ‘family disease’; while this perception leads to delayed treatment initiation because the disease is considered incurable, it also reduces stigma.

Stigma has been well-described in relation to TB [2, 37–41]. Stigma results in difficulties for individuals to accept that they suffer from TB, which delays TB diagnosis. In this study, stigmatisation was mainly reported by vulnerable groups such as drug/alcohol users, migrants, homeless people, daughters-in-law, and ethnic minorities, resulting in further barriers to access health care [42–44]. Stigma from the health staff in hospitals and health centres has a huge impact on the patient’s health seeking and treatment behaviour, as it negatively influences their trust in the health care providers [45].

Other reasons for delays in diagnosis and treatment are the notion that TB is a chronic, inherited, and/or incurable disease and people’s health seeking behaviour, consisting of self-treatment at home and/or the folk sector. This folk sector consists of non-professional specialists outside the official government health system, including all kinds of healers who are not part of the official medical system. Most of them are not full-time healers and in most cases not officially accepted [46]. A study by Stickley et al (2013) in eight countries of the former Soviet Union suggests that since the collapse of the Soviet Union there has been a sharp increase in the use of complementary and alternative medicine in some former Soviet countries [7]. The prevalence of consulting an alternative (folk) medicine practitioner for symptom treatment varied widely between countries, ranging from 3.5% in Armenia to 25.0% in Kyrgyzstan. Aitpaeva et al (2007) and Molchanova (2009) elaborated how folk healing, Islam and the official mental health system coexist in Kyrgyzstan [9, 46]. The attraction of the popular and folk sector lies in the fact that it does not only offer diagnosis and cure, but also an explanation for the illness the person is suffering from. This helps the patient to understand why he or she is sick in order to accept the disease and the treatment [47–49].

DR-TB treatment is among the most painful treatments regarding side effects [50, 51] and duration. Patients are confronted with the feeling that the treatment for DR-TB seems to be even worse than the disease itself. As the title of this paper ‘My favourite day is Sunday’–a statement made by a patient–suggests, Sunday is the day patients prefer because it is the day they do not have to take treatment. Patients form an opinion about their treatment by consciously or subconsciously testing how the medicine works on their body [52]. Understanding the causes of patients’ non-adherence is an important feature in order to know how to deal with non-adherence and to propose supportive actions [39]. However, ‘adherence’ does not entail a patient-centred approach. The terms ‘adherence’ and ‘compliance’ carry an undertone of paternalism and, in the context of a patient’s expected compliant or adherent behaviour, also suggest that the blame lies with the patients when their behaviour does not meet the health care professionals’ recommendations [53]. This study concentrates on the patient perspective of treatment in order to move beyond a ‘compliant’ or ‘adherent’ patient and towards a ‘concordant’ relationship between doctor/nurse and patient. By using the concept of concordance, the need of moving beyond the established terms ‘adherence’ or ‘compliance’ and the underlying assumption of the patient having an ‘irresponsible behaviour’ is signalled [54]. This might be a challenge as in the former Soviet Union it was a commonly held view that it is the individual patient’s responsibility to adhere to treatment and that the patient should bear legal responsibility for non-adherence to treatment and be prosecuted for failure to comply [5].

DR-TB treatment is composed of drugs and psychosocial support. The social workers and the PSEC team allow for a tailored response for each patient. They work closely with the patient and their families and caregivers. This approach has a huge impact on the patient’s and the family’s understanding of the disease, its treatment course, the associated side effects, and the healing process. This is why many patients and families favour an ambulatory model of treatment and care, as similarly described in a study from Uganda [55]. The extent to which the family, community and household support the patient–emotionally and financially–has an important impact on treatment concordance. A positive, non-stigmatising environment is thus another crucial characteristic that supports patients and goes along with the community’s acceptance of their suffering.

One limitation of this study may have been that the respondents knew that the investigators worked for MSF, which may have biased responses. This bias was addressed in part by carefully explaining the role of the anthropologist and her neutrality, and strict assurance of anonymity and confidentiality. Another limiting element of data collection was the fact that people related to the MSF network arranged most of the interviews. However, experiences and opinions of respondents that were not related to MSF were also successfully collected. All identified patients were individuals that who had already been diagnosed with TB or DR-TB, which could be another limitation, as people with specific barriers to access may not have been included. Nevertheless, vulnerable groups with known difficulties in accessing health care facilities were interviewed.

## Recommendations

A number of recommendations can be extracted from our study. First, our results support the call for a change in the language concerning TB: for instance, calling a patient who stopped treatment a ‘defaulter’ holds the patient responsible and puts the blame on the individual [46]. Similarly, our results show that the way language is used to frame a disease (tuberculosis versus ‘cold of the lungs’; the unacceptability of blaming individuals for TB transmission) has an immense impact on the way individuals seek care. Therefore, and to support a patient-centred approach [54], a change in language is encouraged; for example, talking about patients who were ‘not able to start’ treatment and patients who ‘stopped’ treatment, respectively.

Second, health promotion activities should continuously highlight that TB and DR-TB are curable when patients are diagnosed, concur with treatment, and complete treatment. At the same time, efforts should continue to address popular TB concepts such as ‘lungs catching a cold’ and ‘family disease’ and to simultaneously conduct information campaigns on transmission, prevention and symptoms for communities.

Third, trust in health care providers–MoH and MSF–is crucial for patients’ concordance with treatment. For some patients, health care providers are the only contact persons during the treatment course. These health care providers should be trained in the counselling of patients in pain, i.e. in displaying an empathic attitude towards DR-TB patients, and should be made aware of the importance of their role during treatment. Collaboration with *Tabib* should be sought in order to combine classic TB treatment with alternative methods and minimise negative impact on DR-TB treatment. The *Tabib* should be trained on referral and receive education about TB and DR-TB.

Fourth, active case finding is promoted. Patients, together with family members, should be motivated to start treatment. There should be a critical assessment with the patient and the family to determine if ambulatory treatment is the most convenient option. Emotional support through the PSEC team and financial support for transport should be maintained. A shorter treatment course for DR-TB should be implemented as soon as possible.

Fifth, stigma should be tackled; for instance, expert clients (cured DR-TB patients) could be included as positive examples for the communities to emphasize that TB is curable. Effective access of vulnerable groups to diagnosis and treatment should be advocated for, and community awareness strategies adapted to each vulnerable group should be put in place. NGOs working with neglected groups and shelter organisations should be supported in terms of facilitating access to health care for vulnerable groups.

## Conclusion

This study has analysed the perception of TB and DR-TB from the perspective of the general population, patients, caregivers and health care providers (doctors and nurses) in Kara Suu District and Osh city. The perception of TB as the ‘lungs having a cold’ likely delays health seeking and treatment initiation. Poor and neglected people (drug/alcohol users, Luli community, etc.), migrants and young women are most vulnerable in terms of barriers to health services. To address these barriers, a programme focusing on the needs of the population in a culturally sensitive way, with a respectful and empathic attitude, is crucial. The study showed that a decentralised ambulatory model of care supports patients’ emotional state to complete their treatment. Health care providers are of utmost importance, with the support of the social workers. They acknowledge the crucial role they play in accompanying DR-TB patients during the treatment course and recognise a change in their own attitudes–as do the patients and caregivers’. The success of such a decentralised model of care depends on the degree of acceptance reached in the population as well as on the health staff carrying out their work in a stigma-free and supportive fashion.

## Supporting Information

S1 File Literature Review: The following review gives an inclusive overview of current social science and medical literature covering the subjects Kyrgyzstan, health system and tuberculosis, multidrug-resistant tuberculosis (MDR-TB), HIV/AIDS and TB. It is structured in the following chapters:. 1. Kyrgyzstan, general information, politics, ethnic and social groups. 2. National health system/health policy. 3. TB medical articles, multi-drug resistance and TB control. 4. Access to TB treatment. 5. Patient adherence and compliance. 6. Stigma, discrimination, marginalised groups and perception of TB. 7. Local knowledge and believes, attitudes, health seeking behaviour. 8. Health promotion, community based activities and trainings. The articles are ranked by relevance (prioritising articles about Kyrgyzstan and naming experiences and case studies from other countries later) and date of appearance, respectively.
(PDF)
