# Supplementary material for: “My Favourite Day Is Sunday”: Community Perceptions of (Drug-Resistant) Tuberculosis and Ambulatory Tuberculosis Care in Kara Suu District, Osh Province, Kyrgyzstan
Source: PLoS One. 2016 Mar 28;11(3):e0152283. doi: 10.1371/journal.pone.0152283 (PMC4809488; doi:10.1371/journal.pone.0152283)
Supplement: S1 File — It is structured in the following chapters:. 1. Kyrgyzstan, general information, politics, ethnic and social groups. 2. National health system/health policy. 3. TB medical articles, multi-drug resistance and TB control. 4. Access to TB treatment. 5. Patient adherence and compliance. 6. Stigma, discrimination, marginalised groups and perception of TB. 7. Local knowledge and believes, attitudes, health seeking behaviour. 8. Health promotion, community based activities and trainings. The articles are ranked by relevance (prioritising articles about Kyrgyzstan and naming experiences and case studies from other countries later) and date of appearance, respectively. (PDF) [file pone.0152283.s001.pdf]

LITERATURE REVIEW

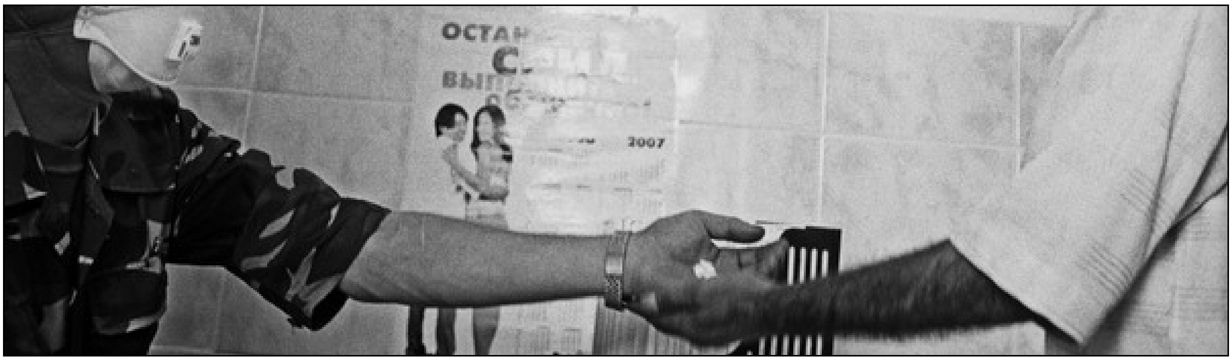

© Alexander Glyadyelov

Multi-drug resistant tuberculosis in Kyrgyzstan

Paul Grohma

2013

Table of contents

Abbreviations 1
Introduction 2
1. Kyrgyzstan, general information, politics, ethnic and social groups 2
2. National health system/health policy 3
3. Tuberculosis medical articles, multi-drug resistance and tuberculosis control 5
4. Access to tuberculosis treatment 6
5. Patient adherence and compliance 7
6. Stigma, discrimination, marginalised groups and perception of tuberculosis 8
7. Local knowledge and believes, attitudes, health seeking behaviour 10
8. Health promotion, community-based activities and trainings 12
9. References and links 13
   Ad 1. Kyrgyzstan, general information, politics, ethnic and social groups 13
   Ad 2. National health system/health policy 13
   Ad 3. Tuberculosis medical articles, multi-drug resistance and tuberculosis control 15
   Ad 4. Access to tuberculosis treatment 16
   Ad 5. Patient adherence and compliance 16
   Ad 6. Stigma, discrimination, marginalised groups and perception of tuberculosis 17
   Ad 7. Local knowledge and believes, attitudes, health seeking behaviour 18
   Ad 8. Health promotion, community-based activities and trainings 19
10. Additional reading 20

Abbreviations

CAAP	Central Asia AIDS Project
CSO	civil society organisation
DOTS	directly observed treatment, short-course
DR-TB	drug-resistant tuberculosis
ECDC	European Centre for Disease Prevention and Control
ERS	European Respiratory Society
GFATM	Global Fund to fight AIDS, Tuberculosis, and Malaria
HCW	health care worker
IDU	injection drug user
IUATLD	International Union against Tuberculosis and Lung Disease
MDR-TB	multidrug-resistant tuberculosis
NSE	needle/syringe exchange
NTP	National Tuberculosis Programme
PAL	Practical Approach to Lung Health
RFB	Rifabutin
RIF	Rifampin
TB	tuberculosis
USAID	United States Agency for International Development
USSR	Union of Soviet Socialist Republics
WHO	World Health Organization
XDR-TB	extensively drug-resistant tuberculosis

Introduction

The following review gives an inclusive overview of current social science and medical literature covering the subjects Kyrgyzstan, health system and tuberculosis, multidrug-resistant tuberculosis (MDR-TB), HIV/AIDS and TB. It is structured in the following chapters:

1. Kyrgyzstan, general information, politics, ethnic and social groups
2. National health system/health policy
3. TB medical articles, multi-drug resistance and TB control
4. Access to TB treatment
5. Patient adherence and compliance
6. Stigma, discrimination, marginalised groups and perception of TB
7. Local knowledge and believes, attitudes, health seeking behaviour
8. Health promotion, community based activities and trainings

The articles are ranked by relevance (prioritising articles about Kyrgyzstan and naming experiences and case studies from other countries later) and date of appearance, respectively.

1. Kyrgyzstan, general information, politics, ethnic and social groups

The *Population and Housing Census of the Kyrgyz Republic of 2009* (O. Abdykalykov et al. 2009) as well as the *Brief Statistical Handbook 2008-2011* (À. Osmonaliev et al. 2012) give an extensive overview of main social and demographic characteristics of the population including several maps and charts of administrative and territorial divisions, population density, number of housing units, ethnic/language groups, gender, education, income groups, etc.

The online article *Kyrgyzstan: Ethnic Pluralism and Political Conflicts* by Vladimir Khanin analyses cultural, ethnic, tribal, religious and economic identities and frictions within the country after the Soviet era and how they influence the political structures. It provides insights in internal power relations, regional differences and dynamics of discrimination/marginalisation of minorities.

The article *What Really Happened in Kyrgyzstan?* by S. Radnitz (2006) is an analysis of the causes of Kyrgyzstan's 'Tulip Revolution' in March 2005 and its implications for post-revolutionary politics. The mass mobilisation was the result of community support for local elites after disputed parliamentary elections. The government was overthrown when an improvised alliance of opposition leaders and business elites unified uncoordinated protests around the country. Localism and informal ties were decisive and have persisted in shaping politics since the revolution.

In her book *Gendering Ethnicity* Handrahan (2002) presents a feminist perspective on the relevance of ethnic and gender identities in Kyrgyzstan. She discusses categories like Western vs. Eastern, cultural differences, modernisation and democratisation (and others) in the context of Kyrgyzstan's recent political developments and US democracy support.

The aim of the paper *Tuberculosis trends in Eastern Europe and the former USSR* (Raviglione et al. 1994) is to assess trends in tuberculosis morbidity and mortality in the countries of Eastern Europe and the former USSR. Data on morbidity and mortality were obtained from reports of the Ministries of Health, a 1992 WHO questionnaire, national tuberculosis associations and other sources. Among the Asian countries of the former USSR, Kazakhstan and Tajikistan reported a lower decline in case rates from

1985 to 1990 than from 1980 to 1985. Kyrgyzstan, Turkmenistan, and Uzbekistan reported increases in notification rates from 1985 to 1990: in Turkmenistan an average 5.5% annual increase of the rate was observed between 1987 and 1991.

2. National health system/health policy

The *Kyrgyzstan: Health system review* (Ibraimova et al. 2011) is a broad analysis of Kyrgyzstan's health status (giving an overview of socio-economic facts, economic and political context) and the national health system (organisation and governance, financing, services, human resources). It shows to what extent Kyrgyzstan has undertaken wide-ranging reforms of its health system in a challenging socio-economic and political context. Major improvements of service delivery have included the introduction of new clinical practice guidelines, improvements in the provision and use of pharmaceuticals, quality improvements in the priority programmes for mother and child health, cardiovascular diseases, tuberculosis and HIV/AIDS, strengthening of public health and improvements in medical education. A 'Community Action for Health' programme was introduced through new village health committees, enhancing health promotion and allowing individuals and communities to take more responsibility for their own health.

The article *Integrating tuberculosis and HIV services in low- and middle-income countries: a systematic review* (Legido-Quigley et al. 2013) is a systematic review of studies describing strategies to facilitate TB and HIV service integration, comparing approaches in different countries including Kyrgyzstan.

The *Policy Assessment Report KYRGYZSTAN* (Teitelbaum et al. 2006) is a policy assessment of the Kyrgyz Republic by USAID addressing the Project HOPE objectives of political support and integration, which derive from the WHO recommended strategy of *"sustained political commitment to increase human and financial resources and make TB control a nation-wide activity and an integral part of the national health system."* The report assesses current situations of TB service delivery – primarily the organisation of service delivery, financing and resources – and explores policies and policy-relevant issues that impact the ability of Kyrgyzstan to maintain a sustainable and effective public sector TB programme. General recommendations are made and the report concludes with recommended policy objectives.

In *Kyrgyzstan: still a regional 'pioneer' in HIV/AIDS or living on its reputation?* Ancker et al. (2013) discuss the development of the HIV/AIDS situation in Kyrgyzstan. In contrast to its neighbours, Kyrgyzstan has long been considered a regional pioneer in its response to the HIV/AIDS epidemic, displaying political will and strong leadership, a timely response, and a multi-sectoral approach to tackle the disease. Yet, this progress has become increasingly difficult to sustain in recent years, as it has been undermined by political and social instability, the reorganisation of the Country Coordinating Committee to fight HIV/AIDS, Tuberculosis and Malaria, the lack of unified mechanisms for data collection, monitoring and evaluation, a high rate of turnover of senior and mid-level staff, stigma and discrimination faced by those most at risk and heavy dependence on external donors.

The study *Feasibility test results of the Practical Approach to Lung Health in Bishkek, Kyrgyzstan* by Brimkulov (2009) assesses the results of training family doctors in Practical Approach to Lung Health (PAL) techniques. Findings suggest that PAL training has resulted in a decrease by one-third in referrals to hospitals, specialists or diagnostic tests. Data do not show any improvement in tuberculosis case detection. However, in the impact survey the number of drugs prescribed per patient decreased by 13.6% and the average cost of prescription of any drug per patient was reduced by 32.2%. The study suggests that training in standardised PAL guidelines is likely to reduce referral as well as drug prescription costs for respiratory patients.

Erhola (2009) reports in the paper *Development process of the Practical Approach to Lung Health in Kyrgyzstan* that the Practical Approach to Lung Health (PAL) strategy was adopted by the Ministry of Health of Kyrgyzstan to improve the quality of case management of priority respiratory illnesses, including tuberculosis. The process of development and implementation of the strategy is described in the present study. The process followed ten steps, which included the government decision to support PAL development and the establishment of a national working group in charge of adapting guidelines, developing training materials and testing the feasibility and impact of the guidelines.

The article *Introducing health insurance: The challenges faced by the Kyrgyz model* by Kasiev et al. (2000) describes the late and difficult establishment of modern health structures including a mandatory health insurance system across Kyrgyzstan. It explains mechanisms like quality management, coverage and provider payments and concludes that health insurance faces a considerable amount of potential risks, including a lack of resources, the emergence of a two-tier system (and the erosion of equity), along with cream-skimming among the population.

The policy brief *The Impact of Global Health Initiatives in Kyrgyzstan* by the Global HIV/AIDS Initiatives Network (2009) shows the effects of two global health initiatives – the Global Fund to fight AIDS, Tuberculosis, and Malaria (GFATM) and the World Bank's Central Asia AIDS Project (CAAP) – at national and sub-national levels, including the effects on HIV/AIDS service scale-up, human resources, access to HIV/AIDS services and coordination.

In *Public health challenges in Kyrgyzstan: developing a new curriculum* O'Brien et al. (2005) explain that public health challenges in Kyrgyzstan are rooted in the social, economic and political conditions that emerged after the collapse of the Soviet Union in 1991. This project report sets out the case for restructuring public health education in Kyrgyzstan. It also explains how a new public health curriculum will equip Kyrgyz students with the knowledge and skills to work effectively with urban and rural communities in this geographically remote region of Central Asia.

The consultancy report *Pharmacy, Family Planning and Infectious disease activities in Kazakhstan and Kyrgyzstan* (Hafner, Pharm 1997) describes the goals and status of the Zdraf-Reforms and the implementation of integrated settings for family planning and treatment of infectious diseases in family group practices. Another goal of the study was the production of an international/generic brand name drug booklet and its dissemination in family group practices.

The World Bank publication *Stopping Tuberculosis in Central Asia, Priorities for Action* by Godinho (2005) contains a chapter on Kyrgyzstan addressing the main challenges of achieving global TB targets, TB epidemiology, TB control and financing of TB control, including a country profile. It gathers vast information on TB facts and activities in Kazakhstan, Kyrgyzstan, Tajikistan and Uzbekistan.

Raviglione et al. (1996) argue in *Tuberculosis control in Kyrgyzstan – an opportunity* that tuberculosis incidence and mortality rates are rising steeply because they reflect economic hardship and the deterioration of health infrastructure. The human and technical resources needed to reverse this trend are on hand but cannot be used effectively without adequate financial input. The cost of controlling tuberculosis now is modest compared with the cost of allowing it to increase.

In the paper *Limited good and limited vision: multidrug-resistant tuberculosis and global health policy* Jim Yong Kim et al. (2005) discuss the logic of 'cost-effectiveness', which international health policy-makers utilised to make the case for treatment of MDR-TB not being feasible in resource-poor settings. The authors state that policies based solely on analyses of cost-effectiveness of specific interventions for individual settings can be short-sighted and will ultimately hinder progress towards effective global TB control, because they do not pay sufficient attention to the social, political, economic, epidemiological and pathophysiological factors influencing the production of health.

The essay by Keshavjee and Farmer (2012) on *Tuberculosis, Drug Resistance, and the History of Modern Medicine* gives an overview of the history of TB research, international TB policies and the creation of subsequent anti-TB drugs over the past decades. It points out that, while gaining control over TB in the West, poorer countries failed to increase their success in fighting the disease. The article seeks to elucidate the reasons for the weak response to drug-resistant tuberculosis by examining the recent history of tuberculosis policy.

The paper by Migliori et al. (1999) *Tuberculosis management in Europe* sums up the recommendations of a task force of the European Respiratory Society (ERS), WHO and the International Union against Tuberculosis and Lung Disease (IUATLD) (Europe Region) to produce guidelines on TB management in Europe specifically designed for specialists treating TB patients. The guidelines are particularly focused on standardised anti-TB treatment, treatment result monitoring and treatment in special situations.

3. Tuberculosis medical articles, multi-drug resistance and tuberculosis control

In *Global Epidemiology of Tuberculosis* Maher and Raviglione (2005) provide an overview of the current scale of the global tuberculosis epidemic. It describes the global tuberculosis situation as measured by reported and estimated cases and deaths. The increasing threats of HIV-related tuberculosis and drug-resistant tuberculosis receive particular attention. There is a brief review of the extent of implementation of effective tuberculosis control using the DOTS strategy. The article ends with a summary of the approaches needed to accelerate progress in global tuberculosis control.

The article *Surveillance, co-infection, resistance: tuberculosis in Europe* (Burki 2011) describes how the WHO and the European Centre for Disease Prevention and Control (ECDC) for the past years have jointly been collecting data on tuberculosis in the 53 countries that make up the WHO European Region. Rates of tuberculosis in the region continued to fall but MDR-TB rates remain high. Treatment success rates, on the other hand, are not high enough. Deaths by TB mainly occurred in the 18 nations identified as high-priority countries; places such as the Baltic states, Bulgaria and Romania in the European Union, as well as Georgia, Kyrgyzstan, and Moldova among the former Soviet republics.

The paper *Clusters of Multidrug-Resistant Mycobacterium tuberculosis Cases, Europe* (Devaux et al. 2009) discusses molecular surveillance of MDR-TB and the detection/analysis of international clusters. Clustering and the Beijing genotype were associated with strains originating in Eastern European countries. Molecular cluster detection contributes to identification of transmission profile, risk factors, and control measures.

In *The Rationale for Using Rifabutin in the Treatment of MDR and XDR Tuberculosis Outbreaks* Sirgel et al. (2013) assess the rationale for using RFB as a substitution for RIF in the treatment of MDR and XDR tuberculosis outbreaks in South Africa. The study's results may assist clinicians and policy makers to make objective therapeutic decisions, especially in situations where therapeutic options are limited.

The book *AIDS and tuberculosis – a deadly liaison* by Kaufmann et al. (2009) concludes that providing timely information on prevention, diagnosis and therapy is the first reference to emphasise the increasing coexistence of these two life-threatening diseases. In various articles the authors discuss recent aspects of HIV-TB interaction, clearly divided into three sections on immunology and vaccination strategies, drugs and clinical issues.

The WHO *Guidelines for management of drug-resistant tuberculosis: emergency update* (2008) provide updated guidelines and recommendations on how to manage drug-resistant TB (DR-TB) based on a rapid assessment of the best available evidence by a group of experts. It serves as guidance for TB control

programmes and medical practitioners on all aspects of the management of DR-TB, including XDR-TB. It also contains a chapter on patient-centred care.

Toman’s tuberculosis – case detection, treatment, monitoring – questions and answers by Frieden (2004) is an updated edition of *Toman’s Tuberculosis* (1979) adding new case materials and newer diagnostic modalities. Sections on appropriate case detection strategies are also included. The treatment section has been updated with information on short-course treatment. Updated information on host defences, drug resistance, drug dosages, extra-pulmonary tuberculosis, treatment adherence and direct observation of treatment has been added. Sections on the basis, role and limitations of treatment for tuberculosis infection have been included as well as a section on monitoring programme effectiveness, largely based on the experience of DOTS implementation in various countries. The recording and reporting system established by Styblo is simple, robust and effective; it serves as the basis for accountability and programme monitoring.

Similarly, *Crofton's Clinical Tuberculosis* by Rieder et al. (2009) is an updated version of the 1993 standard book discussing TB in children, pulmonary and extra-pulmonary TB in adults, TB and HIV/AIDS, treatment of TB, drug use, surgery and preventive treatment.

The book *Priorities for tuberculosis bacteriology services in low-income countries*, second edition, by Rieder et al. (2007) provides useful guidance for national officers responsible for the reference laboratory on its role, main responsibilities and technical and organisational aspects of smear examination and surveillance of anti-tuberculosis drug resistance. It is important to note here that quality assurance and control, training and monitoring are all general functions of the laboratory, and while this book addresses tuberculosis, the same principles are applicable to other diseases or functions.

The Turkish study *Side effects associated with the treatment of multidrug-resistant tuberculosis* by Törün et al. (2005) aims at reporting the frequency of treatment side effects in cases of multidrug-resistant tuberculosis. A retrospective review of the medical records of 263 patients who received individualised treatment for MDR-TB revealed one or more side effects developed in 182 cases (69.2%). The authors conclude that timely and aggressive management of drug side effects means that high side effect rates in MDR-TB treatment need not compromise success rates.

In *Tuberculosis control in the Caucasus: successes and constraints in DOTS implementation* Zalesky et al. (1999) describe the pilot projects for tuberculosis control, supported by the WHO and based on the WHO recommended control strategy directly-observed treatment, short-course in the Caucasian countries (Armenia, Azerbaijan, Georgia) two years after their implementation. An analysis of data on case detection, sputum conversion and treatment outcome by the Ministries of Health in each country reported quarterly to the WHO.

4. Access to tuberculosis treatment

The paper *Exploitation, vulnerability to tuberculosis and access to treatment among Uzbek labour migrants in Kazakhstan* (Huffmann et al. 2012) as well as the Final Report of the Project HOPE *Awareness of Tuberculosis and Access to Health Services and Tuberculosis Treatment among Uzbek Labor Migrants in Kazakhstan* (Huffmann 2009) report findings from qualitative studies conducted with migrants, TB patients and health care workers to understand the mechanisms that impede migrants’ access to care. Findings describe three structural contexts – the employment, legal and health care contexts – which act in concert to render migrants vulnerable to exploitative work conditions and cause a series of barriers to health care. These conditions contribute to increased exposure to TB, heightened risk of reactivation due to weakened immunity, treatment seeking delays and increased severity of disease. Seasonal migration patterns also contribute to treatment interruption, which constitutes a risk

for the creation of drug resistance. Using the theory of structural violence coupled with the concept of cumulative vulnerability, this paper analyses how illegality interacts with exploitation and social marginalisation to produce vulnerability to TB and restrict access to treatment.

In *Patient Adherence to Tuberculosis Treatment: A Systematic Review of Qualitative Research* Munro et al. (2007) explain why poor adherence to treatment is common despite various interventions aimed at improving treatment completion. Lack of a comprehensive and holistic understanding of barriers to and facilitators of treatment adherence is currently a major obstacle to finding effective solutions. The aim of this systematic review of qualitative studies is to understand the factors considered important by patients, caregivers and health care providers in contributing to TB medication adherence.

In her article *Poverty, out-of-pocket payments and access to health care: evidence from Tajikistan* Falkingham (2004) uses the Tajikistan Livings Standard Survey to investigate the level and distribution of out-of-pocket payments for health care in Tajikistan and to examine the extent to which such payments act as barriers to health care access. The data show that there are significant differences in health care utilisation rates across socio-economic groups and that these differences are related to the ability to pay. Official and informal payments are acting both to deter people from seeking medical assistance and, once advice has been sought, from receiving the most appropriate treatment. Despite informal exemptions, out-of-pocket payments for health care are exacting a high toll on household welfare with households being forced to sell assets or go in debt to meet the costs of care. Urgent action is needed to ensure equity in access to health care.

The study *Health service providers' perceptions of barriers to tuberculosis care in Russia* by Dimitrova, B. et al. (2006) explores health care providers' perceptions of existing barriers to access to TB services in urban and rural areas of Samara Oblast in Russia. Barriers were identified in inter-connected areas: barriers associated with the health care system, care process barriers, barriers related to wider contextual issues and barriers associated with patients' personal characteristics and behaviour. In the health care system, insufficient funding was identified as an underlying problem resulting in a decrease in screening coverage, low salaries, staff shortages, irregularities in drug supplies and out-dated infrastructure. Worsening socio-economic conditions were seen both as a cause of TB and a major obstacle to access to care. Behavioural characteristics were identified as an important barrier to effective care and treatment, and health staff favoured compulsory treatment for 'non-compliant' patients as well as involvement of the police in defaulter tracing. TB was profoundly associated with stigma and this resulted in delays in accessing care and barriers to ensuring treatment success.

5. Patient adherence and compliance

Niyi Awofeso (2008) outlines in a short article why *Anti-tuberculosis medication side effects constitute major factor for poor adherence to tuberculosis treatment* among prison inmates in Kyrgyzstan. It reflects upon the local significance of the DOTS approach with its concepts of adherence and compliance.

The qualitative study from Ethiopia *Barriers and facilitators of adherence to TB treatment in patients on concomitant TB and HIV treatment* by Gebremariam et al. (2010) explore patients' and health care professionals' views about barriers to and facilitators of TB treatment adherence in TB/HIV co-infected patients on concomitant treatment for TB and HIV. Factors that influenced adherence to TB treatment positively were beliefs in the curability of TB, beliefs in the severity of TB in the presence of HIV infection and support from families and health professionals. Barriers to treatment adherence were the experience of side effects, pill burden, economic constraints, lack of food, stigma with lack of disclosure and lack of adequate communication with health professionals.

Promoting adherence to treatment for tuberculosis: the importance of direct observation by Frieden and Sbarbaro (2007) describes the major elements of the DOTS strategy as follows: political commitment by governments, improved laboratory services, a continuous supply of good-quality drugs and a reporting system to document the progress (and failure) of treatment for individual patients and of the programme. The fifth element, effective case management via direct observation of treatment by an independent and trained third party, was a response to decades of reports documenting the failure of patients to complete treatment. Simply put: direct observation of treatment is an integral and essential component of DOTS.

S. Thiam et al. (2007) describe in *Effectiveness of a Strategy to Improve Adherence to Tuberculosis Treatment in a Resource-Poor Setting* why poor adherence to treatment remains a major obstacle to efficient TB control in developing countries by the example of Senegal. The study's intervention package, based on improved patients counselling and communication, decentralisation of treatment, patient choice of DOT supporter and reinforcement of supervision activities, led to improvement in patient outcomes compared with the usual TB control procedures. This approach may be generalised in the context of TB control programmes in resource-poor countries.

In M.T. Wright's (2000) study *The old problem of adherence: research on treatment adherence and its relevance for HIV/AIDS* the internationally published research on patient adherence was selectively reviewed with the goal of determining its relevance for the treatment of HIV/AIDS. Results show that not adhering to treatment regimens is so widespread that no combination of socio-demographic variables is reliably predictive of patients not following doctors' orders. Characteristics of the patients' situation, of the given therapy and of the disease itself affect adherence. In addition, the patient-doctor relationship and the context of the treatment are important. Often overlooked are the existential dimensions of meaning, self-determination and quality of life, which are particularly important for the chronically ill. Treatment needs to be negotiated individually with each patient on the basis of an open therapeutic relationship and with the help of multidimensional interventions. Lessons from the discourse on safer sex can steer adherence research and practice away from a behavioural and reductionist approach towards the context and meaning of treatment.

The article *Incentives and accessibility: A pilot study to promote adherence to TB prophylaxis in a high-risk community* (Watters et al., 1999) reports about the success of a community-based directly observed preventive therapy (DOPT) programme for treatment of latent tuberculosis infection among injection drug users (IDUs) in an US inner-city neighbourhood using 'cash incentives', which resulted in high levels of adherence and treatment completion among drug users.

A qualitative study of medication-taking behaviour in primary care by Dowell/Hudson (1997) investigates factors influencing medication intake patterns in general (not TB specific) based on interviews with 44 patients in a UK general hospital. The patients show different disease and treatment backgrounds, and drug users were excluded from the study. Nevertheless, the result of the study is a comprehensive therapeutic decision model, which could be used in other contexts, such as TB medication. It explains the mechanisms of understanding, acceptance, scepticism and rejection within the treatment cycle and proposes new approaches to enhance treatment adherence, safe medication and patient satisfaction.

6. Stigma, discrimination, marginalised groups and perception of tuberculosis

The WHO publication *Drug use in prisons in Kyrgyzstan: a study about the effect of health promotion among prisoners* (Moller et al. 2008) states that in Kyrgyzstan the prevalence of injecting drug behaviour is among the highest found throughout the world. Health promotion training, improved health care and

needle/syringe exchange (NSE) programmes have been shown to decrease risk behaviour among injecting drug users. In Kyrgyzstan, an intervention study with training of prison staff and prisoners was performed in one prison. Before and after the training, a random selection of the prisoners answered a questionnaire about drug use, risk behaviour and health care. The survey was carried out in both the intervention prison and in a reference prison. The number of drug users, the use of drugs and risk behaviour were improved significantly within half a year and especially the injection and use of drugs decreased in the intervention group. The study clearly shows that increased focus, improved health care and training of prisoners and staff on drug use and harm reduction can reduce both use of drugs and risk behaviour.

The paper *Penitentiary population of Mycobacterium tuberculosis in Kyrgyzstan: Exceptionally high prevalence of the Beijing genotype and its Russia-specific subtype* by Mokrousov (2009) presents results of the first study of the Mycobacterium tuberculosis genotypes circulating in Kyrgyzstan. It focuses on the incarcerated population known to be at high-risk for tuberculosis, with a significant impact on TB incidence in the general population. To conclude, all markers taken together the penitentiary population of M. tuberculosis in Kyrgyzstan exhibited a strong genetic affinity to Russia and a weak relatedness to East Asia.

In *Addressing multidrug-resistant tuberculosis in penitentiary hospitals and in the general population of the former Soviet Union* Portaels et al. (1999) point out that in jails in Baku, Azerbaijan and Mariinsk, Siberia the rates of MDR-TB among 'newly enrolled' and 'non-responding' cases were 24.6% and 92.1%, respectively. Studies strongly suggest transmission of MDR-TB between prisoners. There are no coherent guidelines for TB control programmes confronted with high pre-existing levels of MDR-TB but with only limited laboratory, clinical, pharmaceutical and financial resources. A 'DOTS plus' strategy, where an established TB control programme is complemented by facilities to treat MDR-TB patients, has been advocated.

In *HIV and tuberculosis: The construction and management of double stigma* Daftary (2012) states that the mitigation of the tuberculosis (TB) and HIV endemic is undermined by critical clinical, operational and social challenges of which the social aspects have been least explored. This paper examines the lived experience of TB disease and HIV from the perspective of affected individuals to analyse how they may think about their dual illness. Dual illness introduced a paradox to patients' identity constructions and produced a unique, overlapping double stigma. This facilitated new forms of stigma against TB and aggravated existing stigma against HIV.

The article by Courtwright and Turner (2010) *Tuberculosis and Stigmatization: Pathways and Interventions* states that institutional and community norms that lead to stigmatisation of TB are thought to hinder TB control. The authors performed a systematic review of the literature on TB stigma to identify the causes and evaluate the impact of stigma on TB diagnosis and treatment. Several themes emerged: fear of infection is the most common cause of TB stigma; TB stigma has serious socio-economic consequences, particularly for women; qualitative approaches to measuring TB stigma are more commonly utilised than quantitative surveys; TB stigma is perceived to increase TB diagnostic delay and treatment non-compliance, although attempts to quantify its impact have produced mixed results; and interventions exist that may reduce TB stigma.

The importance of addressing the unfolding TB-HIV stigma in high HIV prevalence settings (Bond/Nyblade 2006) stresses that, in the context of high HIV prevalence, tuberculosis is proving hard to control, becoming an increasingly prevalent disease and forcing a more integrated public health approach to the dual epidemics. Yet, this integrated approach often overlooks the implications of how intertwined TB and HIV have become in the social reality of people living in high HIV prevalence settings. The paper demonstrates the strength of the association between TB and HIV in urban and rural Zambia

and how visible signs of TB become triggers for TB-HIV stigma. It explores three key causes of this stigma that could be addressed by integrated TB-HIV services.

The aim of the study by Khan et al. (2006) *Knowledge, Attitude and Misconceptions regarding Tuberculosis in Pakistani Patients* was to assess knowledge of patients with tuberculosis about their disease and misconceptions regarding TB. It showed that misconceptions regarding tuberculosis were widespread in Pakistani patients. Poor knowledge of TB patients concerning their disease may contribute to the high burden of TB disease in the country. The level of knowledge and awareness about TB is known to correlate with seeking health care and time of presentation. By educating the patients and removing their misconceptions, patient compliance with therapy and spread of disease is likely to improve.

In *Perception and Social Consequences of Tuberculosis: A Focus Group Study of Tuberculosis Patients in Sialkot, Pakistan* R. Liefooghe et al. (1995) point out that treatment defaulting is one of the major causes of failure of TB control programmes. A focus group discussion study has been carried out to gain a better understanding of the impact of social stigmatisation, treatment cost and pregnancy on defaulting. The findings of this study reveal the urgent need for a health education campaign to convince the general population that tuberculosis is curable. All health care providers should act as 'de-stigmatisers'.

In the study from New Zeeland *The Perceptions And Beliefs Of Healthcare Workers About Clients With Tuberculosis* Miller (2007) explores attitudes and beliefs of health care workers (HCW) towards clients with TB and the impact of these attitudes and beliefs on care. The beliefs of HCW relating to stigma associated with TB were also reviewed. HCWs reported that TB clients and their families suffer greatly from the impact of TB. These effects include emotional, psychological, physical, practical, social and economic factors. The observed level of impact of TB on clients depended on their identity, which was influenced by their cultural and spiritual beliefs, their level of knowledge and the relationship they had with their HCW.

7. Local knowledge and believes, attitudes, health seeking behaviour

The book *Mazar Worship in Kyrgyzstan: Rituals and Practitioners in Talas* by Aitpaeva et al. (2007) examines the core traditional belief system of the Kyrgyz people: the Mazar ritual and the tradition of worshiping holy Mazar sites. This ancient cultural tradition is still influencing perceptions of illness and traditional healing in Kyrgyzstan today. The first part of the book contains information about the experiences of the cultural practitioners acting as traditional healers, dervishes, clairvoyants and those who recite the Manas epic. The second part of the book contains the thoughts and opinions of scholars from different disciplines who wrote about the sacred sites.

A study by Stickley et al. (2013) *Prevalence and factors associated with the use of alternative (folk) medicine practitioners in 8 countries of the former Soviet Union* suggests that since the collapse of the Soviet Union there has been a sharp growth in the use of complementary and alternative medicine in some former Soviet countries. The prevalence of consulting an alternative (folk) medicine practitioner for symptom treatment varied widely between countries, ranging from 3.5% in Armenia to 25.0% in Kyrgyzstan.

Molchanova (2009) explains in her article *Folk Healing, Spirituality and Official Mental Health Sphere in Kyrgyzstan: The Experience of Living Together Being Different* that Kyrgyzstan is a country where folk healing, Islam and the official mental health system exist together in the society, allowing the people to choose among a variety of professions and methods to solve their different health problems. This article provides a short explanation of the three methods, particularly the folk healing, and the interrelation of the three methods. Problems and solutions for these interrelations are also included.

The paper *Status of Counseling and Psychology in Kyrgyzstan* by Molchanova et al. (2008) is an essay about traditional folk healing and the acceptance of/amalgamation with modern psychology and psychotherapy in Kyrgyzstan. It includes chapters on socio-economic and political data and cultural diversity in the country. The study gives an overview of existing models of counselling and psychological interventions and the local perceptions of mental disease and health seeking behaviour.

The article *Mentally ill or chosen by spirits? 'Shamanic illness' and the revival of Kazakh traditional medicine in post-Soviet Kazakhstan* by Penkala-Gawęcka (2013) discusses spiritual healing in post-Soviet Kazakhstan with reference to changing discourses about 'shamanic illness'. What had traditionally been identified as the call of spirits was seized in the Soviet period by biomedical discourse, which ascribed those symptoms to mental illness. Whereas this attitude also influenced popular understandings of 'shamanic illness' at the time, traditional ideas have been gradually restored in the context of the political and social changes of the 1990s. Penkala-Gawęcka argues that this was induced by multiple interconnected factors, among which are the reappraisal and support of the government for Kazakh 'folk' medicine as a part of the national heritage as well as a favourable attitude to local, traditional forms of religiosity. This allowed for collaboration between doctors and healers in the context of institutionalisation of traditional medicine.

The article by Agboatwalla (2003) *Gender perspectives on knowledge and practices regarding tuberculosis in urban and rural areas in Pakistan* investigates gender differences in knowledge of and attitude towards tuberculosis in urban and rural communities in Sindh province, Pakistan. Knowledge of symptoms was generally deficient, particularly in rural females. Regarding TB prevention, 22.4% of rural and 14.4% of urban males said completing treatment was important; only 9.8% of rural and 7.1% of urban females agreed. The study highlights the need to increase population awareness about TB in Sindh.

The study by Wang et al. (2008) *Gender difference in knowledge of tuberculosis and associated health-care seeking behaviours: a cross-sectional study in a rural area of China* reports that TB detection under the national TB control programme in China follows passive case finding guidelines, which could be influenced by the accessibility of health service and patients' health care seeking behaviours. One intriguing topic is the correlation between men's and women's knowledge of TB and their health care seeking behaviours. The study concludes that TB and DOTS programme were not well known by rural Chinese. Gender issues should be considered to reduce diagnostic delay of TB and improve both men's and women's access to qualified health facilities for TB care. Strengthening awareness of TB and improving the accessibility of health care service is essential in TB control strategy, especially under the current vertical TB.

Ngaka ya setswana, ngaka ya sekgoa or both? Health seeking behaviour in Botswana with pulmonary tuberculosis is the title of the article by Steen and Mazonde (1999). The health seeking behaviour of tuberculosis patients and their beliefs and attitudes with regard to the disease were studied. There is an apparent resemblance between traditional ideas of the disease being caused by pollution (breaking of taboos) and modern theories of spread via germs. TB may be regarded as a 'European disease' or as a 'Tswana disease' and this has implications on health behaviour. Patients who regard TB as a 'Tswana disease' may use modern medicine for symptom relief but traditional medicine to treat what they consider the cause of the disease. More knowledge of patients' health seeking behaviour and perceptions would be useful for health workers. The findings of this study could offer suggestions for improvement in the area of health education.

8. Health promotion, community-based activities and trainings

The comparative study by Niyi Awofeso et al. (2008) *Training of front-line health workers for tuberculosis control: Lessons from Nigeria and Kyrgyzstan* points out that efficient human resources development is vital for facilitating tuberculosis control in developing countries, and appropriate training of front-line staff is an important component of this process. It compares the quality, quantity and distribution of tuberculosis physicians, laboratory staff, community health workers and nurses in Nigeria and Kyrgyzstan and highlights implications for (re)training tuberculosis workers in developing countries.

Understanding and Challenging HIV Stigma – Toolkit for Action by the International Center for Research on Women (ICRW 2009) is an HIV centred toolkit, including a short chapter on TB, written for community leaders, AIDS educators, people living with HIV/TB, health workers, teachers, media workers and others involved in the AIDS field, adopted for use in Cambodia. Its aim is to build awareness and commitment to confront stigma and promote more care and support for people living with HIV/TB. It contains extensive materials for *health promotion* (graphics, pictures, drama, role plays etc.) to tackle all aspects of stigma in community work.

The WHO publication from Ethiopia *Engage-TB: integrating community-based tuberculosis activities into the work of nongovernmental and other civil society organizations: operational guidance* by Getahun et al. (2012) provides operational guidance to NGOs in implementing and scaling-up integrated community-based TB prevention, diagnosis, treatment and care using the ENGAGE-TB approach (situation analysis, enabling environment, guidelines and tools, task identification, monitoring and evaluation, capacity-building). It describes the basic operational principles for effective collaboration between NTPs and NGOs and other CSOs. The principles are aligned with the Stop TB Strategy and are complementary to existing guidelines for engaging all health care providers (including NGOs) in TB prevention and care as part of a public–private mix. This guidance emphasises that NGOs or other CSOs providing facility-based TB services (e.g. hospitals, health centres and clinics) should also implement community-based TB activities using the ENGAGE-TB approach.

9. References and links

Ad 1. Kyrgyzstan, general information, politics, ethnic and social groups

O. Abdykalykov et al. — **Population and Housing Census of the Kyrgyz Republic of 2009**
National Statistical Committee of the Kyrgyz Republic Population and Housing Census Department, Bishkek 2009
http://www.stat.kg

À.Osmonaliev et al. — **Brief Statistical Handbook 2008-2011**
The National Statistical Committee of the Kyrgyz Republic
Bishkek 2012
http://www.stat.kg

V. Khanin — **Kyrgyzstan: Ethnic Pluralism And Political Conflicts**
Cummings Center for Russian and East European Studies
Tel Aviv University, Israel
http://www.ca-c.org/journal/2000/journal_eng/eng03_2000/16.khanin.shtml

S. Radnitz — **What Really Happened in Kyrgyzstan?**
Journal of Democracy, 2006, Vol.17 (2), pp.132-146

L. Handrahan — **Gendering Ethnicity – implications for democracy assistance**
Routledge, New York 2002

M.C. Raviglione et al. — **Tuberculosis trends in Eastern Europe and the former USSR**
Tubercle and Lung Disease; Vol. 75, Issue 6, 1994, pp. 400–416
http://apps.who.int/iris/bitstream/10665/61274/1/WHO_TB_94.176.pdf

Ad 2. National health system/health policy

A. Ibraimova et al. — **Kyrgyzstan: Health system review**
Health systems in transition, 2011, Vol.13 (3), pp.xiii, xv-xx, 1-152
http://www.euro.who.int/__data/assets/pdf_file/0017/142613/e95045.pdf

H. Legido-Quigley et al. — **Integrating tuberculosis and HIV services in low- and middle-income countries: a systematic review**
Tropical Medicine and International Health, Vol. 18, No. 2; pp 199–211, February 2013
http://onlinelibrary.wiley.com/

M. Teitelbaum et al. — **Policy Assessment Report KYRGYZSTAN** for the Central Asian TB Control Partnership; USAID publication 2006
http://pdf.usaid.gov/pdf_docs/PNADP492.pdf

S. Ancker et al. — **Kyrgyzstan: still a regional 'pioneer' in HIV/AIDS or living on its reputation?**
Central Asian Survey, 2013, Vol.32(1), p.66-84

N. Brimkulov	**Feasibility test results of the Practical Approach to Lung Health in Bishkek, Kyrgyzstan**<br>The International Journal of Tuberculosis and Lung Disease 2009, Vol. 13 (4): 533 – 539<br>http://docstore.ingenta.com/cgi-bin/
M. L. Erhola	**Development process of the Practical Approach to Lung Health in Kyrgyzstan**<br>The International Journal of Tuberculosis and Lung Disease 2009, Vol. 13(4): 540-544<br>http://docstore.ingenta.com/cgi-bin/
N. Kasiev et al.	**Introducing health insurance: The challenges faced by the Kyrgyz model**<br>Eurohealth, Vol. 6, No. 1; Special Issue Spring 2000<br>http://www2.lse.ac.uk/LSEHealthAndSocialCare/pdf/eurohealth/vol6no2.pdf
Global HIV/AIDS Initiatives Network	**The Impact of Global Health Initiatives in Kyrgyzstan**<br>Global HIV/AIDS Initiatives Network; Policy Brief, July 2009<br>http://epubs.rcsi.ie/cgi/viewcontent.cgi?article=1005&context=ephmpol
V. O'Brien et al.	**Public health challenges in Kyrgyzstan: developing a new curriculum**<br>Rural and Remote Health 5: 461 (Online) 2005<br>http://www.rrh.org.au
M.C. Raviglione et al.	**Tuberculosis control in Kyrgyzstan – an opportunity**<br>World Health Forum, Volume 17, 1996<br>http://whqlibdoc.who.int/whf/1996/vol17-no1/WHF_1996_17(1)_p85-90.pdf
G. Hafner, B.S. Pharm	**Pharmacy, Family Planning and Infectious disease activities in Kazakhstan and Kyrgyzstan**<br>Zdraf-Reform program report to USAID / ENI / HR / HP; 1997<br>http://pdf.usaid.gov/pdf_docs/PNACC995.pdf
J. Godinho	**Stopping Tuberculosis in Central Asia**<br>Priorities for Action<br>World Bank Working Papers No. 56, 2005<br>https://openknowledge.worldbank.org/bitstream/
Jim Yong Kim et al.	**Limited good and limited vision: multidrug-resistant tuberculosis and global health policy**<br>Social Science & Medicine 61 (2005) 847–859
S. Keshavjee / P.E. Farmer	**Tuberculosis, Drug Resistance, and the History of Modern Medicine**<br>New England Journal of Medicine 367; 10 (2012)<br>http://www.nejm.org/doi/full/10.1056/NEJMra1205429
G.B. Migliori et al.	**Tuberculosis management in Europe**<br>Recommendations of a Task Force of the European Respiratory Society (ERS), the World Health Organisation (WHO) and the International Union against Tuberculosis and Lung Disease (IUATLD) Europe Region; European Respiratory Journal, 1999; 14: 978-992

Ad 3. Tuberculosis medical articles, multi-drug resistance and tuberculosis control

D. Maher, M. Raviglione — **Global Epidemiology of Tuberculosis**
Clin Chest Med 26 (2005), pp. 167 – 182
http://elib.fk.uwks.ac.id/asset/archieve/e-book/ILMU PENYAKIT PARU

T. Burki — **Surveillance, co-infection, resistance: tuberculosis in Europe**
The Lancet Infectious Diseases - Volume 11, Issue 5 (May 2011)

Devaux et al. — **Clusters of Multidrug-Resistant Mycobacterium tuberculosis Cases, Europe**
Emerging Infectious Diseases, 2009, Vol.15 (7), p.1052-1060
http://www.ncbi.nlm.nih.gov/pmc/articles/PMC2744220/

F.A. Sirgel et al. — **The Rationale for Using Rifabutin in the Treatment of MDR and XDR Tuberculosis Outbreaks**
PLoS ONE 2013; 8(3)
http://www.plosone.org/article/info%3Adoi%2F10.1371%2Fjournal.pone.0059414

S. Kaufmann, B. Walker (eds.) — **AIDS and tuberculosis – a deadly liaison;**
Wiley-VHS, Weinheim 2009
http://onlinelibrary.wiley.com/

M. Rich (ed.) — **Guidelines for management of drug-resistant tuberculosis: emergency update 2008**
WHO, Geneva 2008
http://www.tbrieder.org/publications/books_english/who_treatment_mdr.pdf

T. Frieden (ed.) — **Toman's tuberculosis – case detection, treatment, monitoring – questions and answers (2nd edition)**
WHO, Geneva 2004
http://www.tbrieder.org/publications/books_english/toman_2.pdf

H. L. Rieder et al. — **Crofton's Clinical Tuberculosis**
Third edition; International Union Against Tuberculosis and Lung Disease, Teaching Aids at Low Cost, ed. Oxford: Macmillan Education Ltd, 2009
http://www.tbrieder.org/publications/books_english/crofton_clinical.pdf

H. L. Rieder et al. — **Priorities for tuberculosis bacteriology services in low-income countries; Second edition**
Paris: International Union Against Tuberculosis and Lung Disease, 2007
http://www.tbrieder.org/publications/books_english/red_book.pdf

T. Törün, G. Güngör, et al. — **Side effects associated with the treatment of multidrug-resistant tuberculosis**
Department of Pulmonary Diseases, Süreyyapas, Centre for Chest Diseases and Thoracic Surgery, Istanbul, Turkey; 2005
http://docstore.ingenta.com/cgi-bin/

R. Zalesky et al. **Tuberculosis control in the Caucasus: successes and constraints in DOTS implementation**
The International Journal of Tuberculosis and Lung Disease 1999; 3 (5): pp. 394–40
http://docstore.ingenta.com/

Ad 4. Access to tuberculosis treatment

S.A. Huffman et al. **Exploitation, vulnerability to tuberculosis and access to treatment among Uzbek labor migrants in Kazakhstan**
Social Science & Medicine, 2012, Vol.74 (6), pp.864-872

S.A. Huffman **Awareness of Tuberculosis and Access to Health Services and Tuberculosis Treatment among Uzbek Labor Migrants in Kazakhstan**
Final Report of the Project Hope 2009
http://pdf.usaid.gov/pdf_docs/PDACO258.pdf

S. A. Munro et al. **Patient Adherence to Tuberculosis Treatment: A Systematic Review of Qualitative Research**
PLoS Medicine, July 2007, Vol. 4, Issue 7; e238; pp. 1230
http://www.plosmedicine.org

J. Falkingham **Poverty, out-of-pocket payments and access to health care: evidence from Tajikistan**
Social Science & Medicine 58 (2004) 247–258

B. Dimitrova **Health service providers' perceptions of barriers to tuberculosis care in Russia**
Health Policy Plan 2006; 21(4); pp.: 265 - 74
http://heapol.oxfordjournals.org/content/21/4/265.long

Ad 5. Patient adherence and compliance

N. Awofeso **Anti-tuberculosis medication side-effects constitute major factor for poor adherence to tuberculosis treatment**
Bulletin of the World Health Organisation, Vol. 86, N. 3, 2008
http://www.scielosp.org/scielo.php

M. Gebremariam et al. **Barriers and facilitators of adherence to TB treatment in patients on concomitant TB and HIV treatment: a qualitative study**
BMC Public Health 2010, 10:651
http://www.biomedcentral.com/1471-2458/10/651

T. R. Frieden, J. A. Sbarbaro **Promoting adherence to treatment for tuberculosis: the importance of direct observation**
Bulletin of the World Health Organ Vol. 85, No. 5, 2007
http://dx.doi.org/10.1590/S0042-96862007000500023

S. Thiam et al.	**Effectiveness of a Strategy to Improve Adherence to Tuberculosis Treatment in a Resource-Poor Setting: A Cluster Randomized Controlled Trial**<br>The Journal of the American Medical Association 2007; 297 (4): pp. 380-386<br>http://jama.jamanetwork.com/article.aspx?articleid=205285
Z. Matebesi, C. Timmerman	**The TB patient: qualitative evidence of perceived factors affecting treatment compliance**<br>Centre for Health Systems Research & Development, University of the Free State, Bloemfontein, SOUTH AFRICA; 2003<br>http://humanities.ufs.ac.za/dl/userfiles/Documents/00000/196_eng.pdf
M.T. Wright	**The old problem of adherence: research on treatment adherence and its relevance for HIV/AIDS**<br>AIDS CARE (2000), Vol. 12, Nr. 6, pp. 703–710<br>http://dx.doi.org/10.1080/09540120020014237
J. K. Watters et al.	**Incentives and accessibility: A pilot study to promote adherence to TB prophylaxis in a high-risk community**<br>Journal of Urban Health, 1999, Volume 76, Issue 4, pp 461-467<br>http://www.ncbi.nlm.nih.gov/pmc/articles.pdf
J. Dowell, H. Hudson	**A qualitative study of medication-taking behaviour in primary care**<br>Oxford University Press; Family Practice 1997; Vol. 14, No.5: 369-375<br>http://fampra.oxfordjournals.org/content/14/5/369.long

Ad 6. Stigma, discrimination, marginalised groups and perception of tuberculosis

Moller et al.	**Drug use in prisons in Kyrgyzstan: a study about the effect of health promotion among prisoners**<br>International Journal of Prison Health 2008; 4(3): pp. 124-33
I. Mokrousov et al.	**Penitentiary population of Mycobacterium tuberculosis in Kyrgyzstan: Exceptionally high prevalence of the Beijing genotype and its Russia-specific subtype**<br>Infection, Genetics and Evolution, Vol. 9, Issue 6, 2009, pp. 1400–1405
F. Portaels et al.	**Addressing multidrug-resistant tuberculosis in penitentiary hospitals and in the general population of the former Soviet Union**<br>International Journal For Tuberculosis And Lung Disease, 1999; 3 (7): 582–588
A. Daftary	**HIV and tuberculosis: The construction and management of double stigma**<br>Social Science & Medicine 74 (2012); pp.1512-1519<br>http://www.kit.nl/kit/HIV-and-tuberculosis
A. Courtwright, A.N. Turner	**Tuberculosis and Stigmatization: Pathways and Interventions**<br>Public Health Rep. 2010; 125 (Suppl. 4): 34–42<br>http://www.ncbi.nlm.nih.gov/pmc/articles/PMC2882973/

V. Bond/L. Nyblade **The importance of addressing the unfolding TB-HIV stigma in high HIV prevalence settings**
Journal of Community & Applied Social Psychology; Vol. 16, Issue 6, pp. 452–461 (2006)

J.A. Khan et al. **Knowledge, Attitude and Misconceptions regarding Tuberculosis in Pakistani Patients**
Journal of Pakistan Medical Association, May 2006
http://jpma.org.pk/full_article_text.php?article_id=695

R. Liefooghe et al. **Perception And Social Consequences Of Tuberculosis: A Focus Group Study Of Tuberculosis Patients In Sialkot, Pakistan**
Social Science & Medicine, Vol. 41, Issue 12 (1995); pp. 1685–1692

J.A. Miller **The Perceptions And Beliefs Of Healthcare Workers About Clients With Tuberculosis**
Dissertation, University of Auckland, 2007
http://www.arts.auckland.ac.nz/miller.pdf

Ad 7. Local knowledge and believes, attitudes, health seeking behaviour

G. Aitpaeva et al. **Mazar Worship in Kyrgyzstan: Rituals and Practitioners in Talas**
Aigine Research Center, Bishkek 2007
http://cultureasia2008.cscsarchive.org/pdf/mazar-worship.pdf

A. Stickley et al. **Prevalence and factors associated with the use of alternative (folk) medicine practitioners in 8 countries of the former Soviet Union**
BMC Complementary and Alternative Medicine 2013, 13:83
http://www.biomedcentral.com/1472-6882/13/83

E. Molchanova **Folk Healing, Spirituality and Official Mental Health Sphere in Kyrgyzstan: The Experience of Living Together Being Different**
Journal of Transpersonal Research, Vol. 1, 2009
http://www.transpersonaljournal.com/pdf/vol1-issue2/Molchanova Elena.pdf

E.S. Molchanova et al. **Status of Counseling and Psychology in Kyrgyzstan**
American University in Central Asia; AUCA Academic Review 2008
http://elibrary.auca.kg:8080/dspace/Molchanova etc_2008_1.pdf

D. Penkala-Gawęcka **Mentally ill or chosen by spirits? 'Shamanic illness' and the revival of Kazakh traditional medicine in post-Soviet Kazakhstan**
Central Asian Survey, Vol. 32, Issue 1, 2013 (Special Issue: Focus on Health and Healing in Central Asia) pp. 37-51

M. Agboatwalla **Gender perspectives on knowledge and practices regarding tuberculosis in urban and rural areas in Pakistan**
La Revue de Santé de la Méditerranée orientale, Vol. 9, No 4, 2003
http://www.hope-ngo.com/Portals/0/PDF/genderperspectiveReport.pdf

J. Wang et al. **Gender difference in knowledge of tuberculosis and associated health-care seeking behaviors: a cross-sectional study in a rural area of China**
BMC Public Health 2008, 8:354
http://www.biomedcentral.com/1471-2458/8/354

T.W. Steen, G.N. Mazonde **Ngaka ya setswana, ngaka ya sekgoa or both? Health seeking behaviour in Botswana with pulmonary tuberculosis**
Social Science & Medicine 48 (1999); pp. 163-172
http://www.biomedcentral.com/1472-6963/9/196

Ad 8. Health promotion, community-based activities and trainings

N. Awofeso et al. **Training of front-line health workers for tuberculosis control: Lessons from Nigeria and Kyrgyzstan**
Human Resources for Health 2008, 6:20
http://www.human-resources-health.com/content/6/1/20

ICRW **Understanding and Challenging HIV Stigma – Toolkit for Action**
International Center for Research on Women (ICRW)
http://www.icrw.org/files/publications/Understanding-and-Challenging-HIV-Stigma-Toolkit-for-Action-Cambodia.pdf

H. Getahun et al. **Engage-TB: integrating community-based tuberculosis activities into the work of nongovernmental and other civil society organizations: operational guidance**
WHO Library Cataloguing-in-Publication Data; WHO, Geneva 2012
http://www.who.int/tb/publications/2012/engage_tb_policy/en/index.html

10. Additional reading

A graphical study of tuberculosis incidence and trends in the WHO's European region (1980–2006); Martín Ríos, Toni Monleón-Getino; 2009

Adherence to Long-term Therapies - Evidence for Action; WHO 2003

Causes and Consequences of Human Migration/An Evolutionary Perspective; Crawford & Campbell; Cambridge 2012 (eds.)

Effective tuberculosis control and health sector reforms in Kenya: challenges of an increasing tuberculosis burden and opportunities through reform [Country Case Report]; Hanson & Kibuga; 2000

Emergence and Spread of Extensively and Totally Drug-Resistant Tuberculosis, South Africa; Klopper & Warren et al. 2013

Evolution of Tuberculosis Control and Prospects for Reducing Tuberculosis Incidence, Prevalence, and Deaths Globally; Africa and Eastern Europe; Dye et al. 2005

Health Systems and the Challenge of Communicable Diseases/ Experiences from Europe and Latin America; Coker, Atun, McKee; WHO 2008

Health-system strengthening and tuberculosis control; Cambodia, Tanzania; Atun et al. 2010

High Prevalence of Primary Multidrug Resistant Tuberculosis in Persons with No Known Risk Factors; Peru; Otero et al. 2011

HIV in Central Asia/ Tajikistan, Uzbekistan and Kyrgyzstan; Wolfe et al. in: Public Health Aspects of HIV/AIDS in Low and Middle Income Countries; Springer 2009

Household screening and multidrug-resistant tuberculosis; South Africa; Cox & Cutsern 2011

Interventions for promoting adherence to tuberculosis management; Volmink & Garner 2005

Low access to a highly effective therapy/ a challenge for international tuberculosis control; Dye et al. 2002

Management of MDR-TB/ A Field Guide - A Companion Document to Guidelines for Programmatic Management of Drug-resistant Tuberculosis; WHO 2009

Multidrug-resistant and extensively drug-resistant tuberculosis/ a threat to global control of tuberculosis; South Africa; Gandhi 2010

Occurrence of serious adverse effects in patients receiving community-based therapy for multidrug-resistant tuberculosis; Peru; Furin et al. 2011

Prevalence of and risk factors for resistance to second-line drugs in people with multidrug-resistant tuberculosis in eight countries: a prospective cohort study; 4 articles on Russian Federation; Dalton 2012

Progress towards tuberculosis elimination; Cuba; Gonzales et al. 2007

Resistance to second-line drugs in multidrug-resistant tuberculosis; India; Udwadia 2013

Scratching the surface of ignorance on MDR tuberculosis; Netherlands, USA; Borgdorff & Small 2009

Social change and HIV in the former USSR/ the making of a new epidemic; Atlanti et al. 2006

Spread of Extensively Drug-Resistant Tuberculosis in KwaZulu-Natal Province, South Africa; Moodley et al. 2011

The importance of addressing the unfolding TB-HIV stigma in high HIV prevalence settings; Zambia; Bond & Nyblade 2006

The Rationale for Using Rifabutin in the Treatment of MDR and XDR Tuberculosis Outbreaks; South Africa; Sirgel et al. 2013

Treatment of multidrug-resistant tuberculosis- evidence and controversies; Caminero 2006

Treatment of Multidrug-Resistant Tuberculosis; Iseman 1993 (NEJM)

Tuberculosis burden in households of patients with multidrug-resist and extensively drug-resistant tuberculosis: a retrospective cohort study; Peru; Becerra et al. 2011

Tuberculosis control in the era of HIV; Nunn et al. 2005

Tuberculosis—from ancient plague to modern-day nemesis; The Lancet Vol. 380, 2012

Tuberculosis–HIV co-infection/policy and epidemiology in 25 countries in the WHO European region - Understanding and Challenging Stigma; Lazarus et al. (eds.) 2008

Unexpected high levels of multidrug-resistant tuberculosis present new challenges for tuberculosis control; Hoffner 2012

Unravelling the contexts of stigma - from internalisation to resistance to change; Campbell & Deacon in: Journal of Community & Applied Social Psychology; Special Issue: Understanding and Challenging Stigma 2006
